# Routing Strategies for Isochronal-Evolution Random Matching Network

**DOI:** 10.3390/e25020363

**Published:** 2023-02-16

**Authors:** Weicheng Lun, Qun Li, Zhi Zhu, Can Zhang

**Affiliations:** College of Systems Engineering, National University of Defense Technology, Changsha 410073, China

**Keywords:** isochronal-evolution random matching network, routing strategy, traffic dynamics, path planning, complex dynamic network

## Abstract

In order to abstract away a network model from some real-world networks, such as navigation satellite networks and mobile call networks, we proposed an Isochronal-Evolution Random Matching Network (IERMN) model. An IERMN is a dynamic network that evolves isochronally and has a collection of edges that are pairwise disjoint at any point in time. We then investigated the traffic dynamics in IERMNs whose main research topic is packet transmission. When a vertex of an IERMN plans a path for a packet, it is permitted to delay the sending of the packet to make the path shorter. We designed a routing decision-making algorithm for vertices based on replanning. Since the IERMN has a specific topology, we developed two suitable routing strategies: the Least Delay Path with Minimum Hop (LDPMH) routing strategy and the Least Hop Path with Minimum Delay (LHPMD) routing strategy. An LDPMH is planned by a binary search tree and an LHPMD is planned by an ordered tree. The simulation results show that the LHPMD routing strategy outperformed the LDPMH routing strategy in terms of the critical packet generation rate, number of delivered packets, packet delivery ratio, and average posterior path lengths.

## 1. Introduction

### 1.1. Background

Almost all complex networks are evolving constantly [1], which means that the vertices and edges of the networks are always changing over time [2]. Hence, research on complex dynamic networks is of great theoretical and practical significance. The complex dynamic network has become a functional tool to describe most real networks [3]. This paper focuses on complex dynamic networks that evolve at equal time intervals. Such networks are called the Isochronal-Evolution Networks (IENs). The unit time interval for the evolution of an IEN is called the slot. The topology of the IEN remains unchanged in any slot.

A well-known example of the IEN is Navigation Satellite Networks (NSNs), for example, the Global Positioning System in the USA [4], the Galileo Satellite Navigation System in Europe [5], and the BeiDou Navigation Satellite System (BDS) in China [6]. The Time Division Multiple Access is a key characteristic of IENs [7], which is described as follows. The system period of an NSN, which is the least common multiple of all satellites’ orbit periods and the Earth’s rotation period, is divided into a number of slots. Different Inter-Satellite Links (ISLs) are established between navigation satellites in different slots. An NSN is a type of network whose vertices are satellites and edges are ISLs.

Another characteristic of IENs is that each satellite establishes, at most, one ISL in any slot, because it has only one onboard antenna. Besides IENs, there are many dynamic networks whose edges are pairwise disjoint at any time, such as the Mobile Call Network (MCN). The vertices of an MCN are people with mobile phones. When someone phones another person, an edge is created to connect these two vertices. The edges of an MCN are always pairwise disjoint, because a mobile phone is able to link to, at most, one mobile phone at one time. The MCN is also an IEN with the slot being 1 s. Although there have been some studies on MCNs [8,9,10], very few studies have paid attention to the fact that any two edges of the MCN have no vertex in common.

### 1.2. Related Works on Models for Real-World Networks

Researchers have tried to build mathematical models for various types of real-world networks [11]. In [12], a model of face-to-face interaction networks was developed. In [13], a method was used to generate static networks with edge dynamics. In [14], an activity-driven model of time-varying networks was proposed. In [15], a more statistics-oriented model of communication in social networks was put forward. In [16], a model where both nodes and links are activated by temporal effects was proposed. In [17], a temporal network of the Chinese venture capital market was studied. However, these network models are not suitable for the modeling of a network that evolves isochronally and has a collection of edges that are pairwise disjoint at any time; the reasons for this are as follows.

1. These models do not consider cases where the edges of the aforementioned network are pairwise disjoint at any time.
2. The lifetime of each edge of the aforementioned network may not be continuous and follow a certain probability distribution, which does not meet the conditions of these models.
3. The time scale of the aforementioned network is different from those of these models, because the aforementioned network pays more attention to the value of all slots than to the duration of a slot.

Therefore, we studied how to abstract away a network model from the aforementioned network and investigate it. To the best of the authors’ knowledge, this is the first time that a new complex network model has been proposed to represent NSNs. It is also the first time that a study has focused on the fact that the edges of an MCN are pairwise disjoint at any time.

### 1.3. Model of the Isochronal-Evolution Random Matching Network

We define a matching network as a network in which no two edges have a vertex in common. Matching is a concept used in graph theory to describe a collection of edges of a graph that are pairwise disjoint [18]. The matching network can be modeled using the classic random graph model, which is described as follows.

Given Θ labeled vertices $v_{1}$, $v_{2}$, …, $v_{\Theta }$, where Θ is an even number and Θ>2, we first choose two vertices among these Θ vertices at random to create an edge by connecting them; then, we choose two vertices among the remaining Θ−2 vertices at random to create another edge by connecting them; we continue this process until θ edges have been chosen. Consequently, we generate a matching network with Θ vertices and θ edges, which is called the Random Matching Network (RMN). If an RMN with Θ vertices has $\frac{1}{2}\Theta$ edges, i.e., all vertices are covered, it is known as a Random Perfect-Matching Network (RPMN).

In this paper, an RMN with Θ vertices and θ edges is called RMN(Θ,θ). Obviously, RMN(Θ,θ) is a specific type of Erdos–Renyi random graph with Θ vertices and θ edges [19]. We define an Isochronal-Evolution Random Matching Network (IERMN) as a dynamic network whose topology is an RMN in each slot. Accordingly, a dynamic network whose topology is an RPMN in each slot is known as a Isochronal-Evolution Random Perfect-Matching Network (IERPMN).

### 1.4. Related Works on Routing Strategies

In this paper, we focus on the routing strategy of IERMNs, which is a traffic dynamics problem. Traffic dynamics is a hot research field related to complex networks, in which the main research topic is packet transmission.

In [20,21], the traffic dynamics on multilayer network models were studied, and the impacts of different network structures on the traffic capacity were analyzed. In [22], the impact of the community structure on traffic dynamics in homogeneous random networks was investigated. In [23], the traffic capacity of a network was found to be positively correlated to the entropy of the communication sequence. In [24], the influences of the coupling mode and its corresponding routing strategy on the traffic capacity of the two-layer network were studied. In [25,26], the traffic dynamics in finite buffer networks were studied and a routing strategy motivated by a heuristic algorithm and a routing strategy based on dynamic local information were proposed. In [27], the mechanism by which the network structure impacts traffic dynamics was studied, and a high-contraction-centrality-first strategy was proposed to optimize the network structure.

These existing works focused on the traffic dynamics in static networks rather than dynamic networks. We borrow some useful ideas and methodologies from them but cannot apply their proposed methods in the IERMN.

The remaining of the paper is structured as follows: In Section 2, we introduce the IERMN and the traffic dynamics in the IERMN briefly. In Section 3, we discuss path planning and routing decision-making, which are fundamental concepts of the TD-IERMN. In Section 4, two routing strategies are proposed. In Section 5, the two proposed routing strategies are compared through simulation experiments.

## 2. Isochronal-Evolution Random Matching Network

### 2.1. Introduction of IERMN

The IERMN from $T_{\mathrm{begin}}$ to $T_{\mathrm{end}}$ is designed used the 4-tuple shown in (1)

(1) $$\langle V,\Omega ,\Delta T_{\mathrm{slot}},\theta (s),X\rangle$$

where $\Delta T_{\mathrm{slot}}$ denotes the length of a slot; $\Omega =\frac{T_{\mathrm{end}}-T_{\mathrm{begin}}}{\Delta T_{\mathrm{slot}}}$ denotes the number of slots; θ(s) denotes the function that represents the number of edges in the slot s; $X={\left(x_{ki}\right)}_{\Omega \times \Theta }$ is the matrix that reflects the changes in the edges from slot 1 to slot Ω, and its element $\begin{array}{c} x_{ki} \end{array}$ is defined as (2).

(2) $$x_{ki}=\left\{\begin{array}{ll} j, & v_{i}\;\mathrm{connects}\;\mathrm{with}\;v_{j}\;\mathrm{in}\;\mathrm{slot}\;k\\ i, & v_{i}\;\mathrm{connects}\;\mathrm{with}\;\mathrm{no}\;\mathrm{vertex}\;\mathrm{in}\;\mathrm{slot}\;k \end{array}\right.$$

The IERMN in slot k ($k\in N_{+}$) is denoted by $G_{k}=(V,E_{k})$, where $V=\{v_{i}\}$ is the vertex set, and $E_{k}$ is the edge set. The expression of $E_{k}$ is

(3) $$E_{k}=\{e_{ij}^{\left[k\right]}|v_{i},v_{j}\in V,i<j\}$$

where $e_{ij}^{\left[k\right]}$ means that there is an edge between $v_{i}$ and $v_{j}$ in slot k. The inequality i<j in (3) does not indicate that IERMN is an undirected network (we explain this in Section 2.2). Thus, $e_{ij}^{\left[k\right]}$ does not mean that the direction of this edge is from $v_{i}$ to $v_{j}$. The vertex with a smaller number is called the left vertex, while the vertex with a bigger number is called the right vertex.

We count the number of possible topologies of $G_{k}$ with Θ vertices and θ edges, i.e., the total number of RMN(Θ,θ).

Since edge sets are the differences between different RMN(Θ,θ), the number of RMN(Θ,θ) is just the number of edge sets with θ edges. We can use two steps to generate the edge set of RMN(Θ,θ), which is distinct from the process presented in Section 1.

First step: Select 2θ vertices from Θ vertices. The number of possible selections is the number of 2θ-subsets in a Θ-element set, which is

(4) $$\left(\begin{array}{c} \Theta \\ 2\theta \end{array}\right)=\frac{\Theta !}{(2\theta )!(\Theta -2\theta )!}$$

Second step: Partition the set of 2θ vertices into θ sets, each of size 2. This is a partition problem [28] that can be described as follows. Let n and m be positive integers and let $n_{1}$, $n_{2}$, …, $n_{m}$ be positive integers with $n_{1}=n_{2}=\cdots =n_{m}=\frac{n}{m}$. The number of ways to partition a set of n objects into m unlabeled boxes in which Box r contains $n_{r}$ objects (r∈{1,2,⋯,m}) equals

(5) $$\frac{n!}{m!n_{1}!n_{2}!\cdots n_{m}!}$$

By substituting n=2θ, m=θ, and $n_{1}=n_{2}=\cdots =n_{m}=2$ into (5), we obtain

(6) $$\frac{n!}{m!n_{1}!n_{2}!\cdots n_{m}!}=\frac{(2\theta )!}{\theta !\prod_{r=1}^{\theta }\left(2!\right)}$$

Finally, multiply (4) by (6) to show that the total number of RMN(Θ,θ) is

(7) $$\frac{\Theta !}{\theta !(\Theta -2\theta )!2^{\theta }}$$

Furthermore, by substituting $\theta =\frac{\Theta }{2}$ into (7), we show that the number of RPMN with Θ vertices and $\frac{\Theta }{2}$ edges is

(8) $$\Theta (\Theta -1)\cdots (\frac{\Theta }{2}+1)(2^{-\frac{\Theta }{2}})$$

### 2.2. Introduction of Traffic Dynamics in IERMN

The basic time unit for packet transmission is also the slot. There is two-way alternate communication between two vertices connected by an edge. That is, a slot is divided into two parts equally, and the left vertex sends the packet to the right vertex in the former half slot, while the right vertex sends the packet to the left vertex in the latter half slot. Each half of a slot represents a time step. Therefore, an IERMN is not an undirected network, and the directions of its edges change over time. When a packet is transmitted between two vertices, the vertex that sends the packet is called the predecessor vertex, and the vertex that receives a packet is called the successor vertex.

A packet has fields other than data fields. There are several fields with respect to traffic dynamics, such as the Source Vertex Field (SVF), the Destination Vertex Field (DVF), the Set of Visited Vertices Field (SVVF), and the Future Path Field (FPF). The SVVF and the FPF are introduced in Section 3.3

A vertex of an IERMN is set as a host [29] and a router simultaneously, and it is able to generate, send, and receive packets. Each vertex has a queue to store the packets which are newly created or wait to be sent by this vertex.

The traffic dynamics model of the IERMN can be described as follows [30]:

1. At each time step, R packets with random SVF and DVF are generated in the IERMN, and R is called the packet generation rate.
2. Once a packet has been generated, it is stored in the queue of its source vertex. When a packet is transmitted, it is stored in the queue of the vertex, which is not its destination. If a packet has been delivered to its destination, it will be deleted permanently.
3. At each time step, each vertex can send, at most, C packets ($C\in N_{+}$) to its successor, and C represents the delivery capability of a vertex.

We make the following assumption about the TD-IERMN:

1. A successor vertex receives any packets sent by its predecessor vertex.
2. The queue of each vertex is infinite.
3. Each packet can be transmitted from a predecessor vertex to its successor vertex in one slot.

There is a critical packet generation rate $R_{C}$ for an IERMN. Once $R=R_{C}$, a phase transition takes place from the free-flow state to the congestion state in the IERMN [20]. The free-flow state means that, when $R<R_{C}$, the numbers of generated and delivered packets are balanced, and there is a steady traffic flow in the IERMN. The congestion state means that, when $R>R_{C}$, the newly generated packets overwhelm the delivered packets, and the number of accumulated packets increases with time. The critical packet generation rate $R_{c}$ reflects the maximum traffic capability of an IERMN [31].

The following order parameter H is used to characterize the phase transition [32]:

(9) $$H(R)=\underset{t\to \infty }{\lim}\frac{C}{R}\frac{\langle \Delta W\rangle }{\Delta t}$$

where ΔW=W(t+Δt)−W(t), $\langle \Delta W\rangle$ indicates the average value over a time window Δt, and W(t) represents the number of packets in the IERMN at time step t. When H=0, IERMN is in the free-flow state and $R<R_{C}$; whereas, when H>0, IERMN is in the congestion state and $R>R_{C}$. The larger H is, the worse the congestion is.

## 3. Path Planning and Routing Decision-Making in the IERMN

### 3.1. Definition and Expression of the Path

We define the path in an IERMN as a space–time sequence of the vertices and edges that a packet travels through when it is transmitted from its source to its destination. There are two kinds of paths. The path that is determined before transmitting a packet is called the prior path, while the path that is formed during the transmission process of the packet is called the posterior path.

The path that is from $v_{i}$ to $v_{j}$ and planned at slot k is

(10) $$P_{ijk}=\{(v_{i},e_{i\mu }^{\left[\upsilon \right]},v_{\mu }),\cdots ,(v_{\rho },e_{\rho j}^{\left[ο\right]},v_{j})\}$$

where $\left(v_{i},e_{i\mu }^{\left[\upsilon \right]},v_{\mu }\right)$ denotes one hop of the path, υ is the slot at which the packet departs from its source vertex (so υ is known as the departure slot), and ο is the slot at which the packet arrives at its destination vertex (so ο is known as the arrival slot). For $P_{ijk}$, slot k is known as the planning slot; its departure slot and arrival slot are denoted by $\upsilon (P_{ijk})$ and $ο(P_{ijk})$, respectively.

As a space–time sequence, a path has not only a spatial length but also a temporal length. The temporal length of a path is the number of slots between its departure slot and arrival slot; the spatial length is the number of hops in the path. For $P_{ijk}$, the spatial length and temporal length are denoted by $SL_{ijk}$ and $TL_{ijk}$ respectively. The other meaning of the temporal length is the delay of a path that is counted by slots.

### 3.2. Path Planning

**Figure 1:**
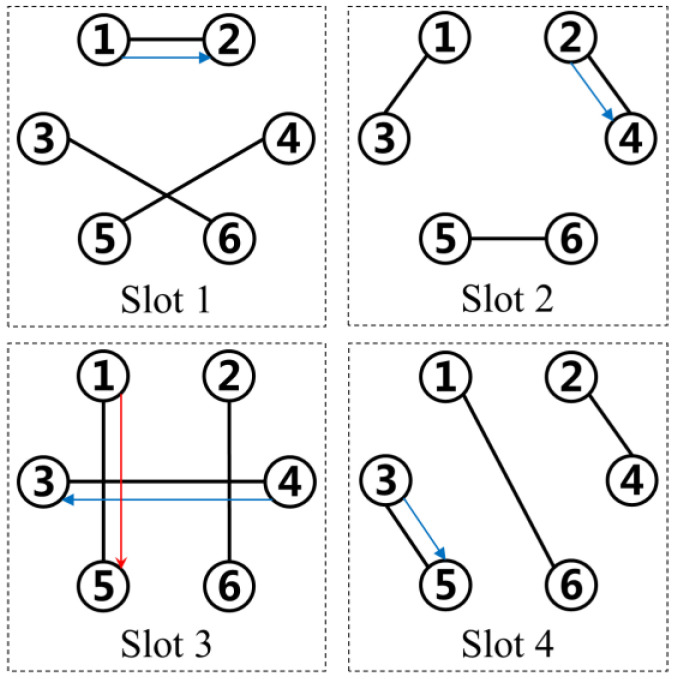
Comparison of two paths. The blue arrows indicate the paths in which vertices are forbidden from delaying the sending of packets. The red arrows indicate the paths in which vertices are permitted to delay the sending of packets.

Path planning is done to design a prior path for a packet before its transmission. In previous studies, a packet was always supposed to keep moving among several vertices until reaching its destination and it was believed to be abnormal for the packet to stay at any vertices. However, in an IERMN, a packet is permitted to stay at some vertices, which is normal and helpful. That is, some vertices delay the sending of the packet in order to make the path shorter. For example, in the IERMN shown in Figure 1, if the vertices are forbidden to delay the sending of packets, we get

(11) $$P_{1,5,1}=\{(v_{1},e_{1,2}^{\left[1\right]},v_{2}),(v_{2},e_{2,4}^{\left[2\right]},v_{4}),(v_{4},e_{3,4}^{\left[3\right]},v_{3}),(v_{3},e_{3,5}^{\left[4\right]},v_{5})\}$$

Both the temporal length and the spatial length of $P_{1,5,1}$ in (11) are 4. However, if the vertices are allowed to delay the sending of packets, we obtain

(12) $$P_{1,5,1}=\{(v_{1},e_{1,5}^{\left[3\right]},v_{5})\}$$

Both the temporal length and the spatial length of $P_{1,5,1}$ in (12) are 1. Therefore, when we study path planning in the IERMN, we give permission for each vertex to delay the sending of packets if necessary.

Although the range of slots for an IERMN is $N_{+}$, paths should be planned with finite slots to decrease unnecessary calculations and data redundancy and to lower the lengths of the paths. Given the planning slot k, slot k+Γ is set to be the latest time to plan paths and Γ represents the planning time window ($\Gamma \in N_{+}$).

### 3.3. Routing Decision-Making

In the process of packet transmission, routing decision-making refers to the selection of routing actions by predecessor vertices. There are two routing actions taken by a predecessor vertex for the packets in its queue: send or hold. Holding a packet means that its sending is delayed, as mentioned in Section 3.2. There are two disciplines for each node:

1. First-in-first-out (FIFO). The packet that is created or received by a vertex is placed at the end of its queue. The vertex always chooses to send the packet that is at the head of the queue.
2. Path iteration avoidance (PIA). Any edge or vertex cannot be visited more than twice by the same packet.

A predecessor vertex makes a routing decision for a packet according to its SVF, DVF, SVVF, and FPF, as mentioned in Section 2.2. The SVVF is a set of all vertices that the packet has visited from its generation to the current slot. The FPF is the path used to transmit the packet in the future. It is assumed that a packet is generated in slot s, and the initial value of its FPF is $P_{SVF,\;DVF,\;s}$.

The routing decision-making algorithm used for the predecessor vertices is shown in Algorithm 1. In Algorithm 1, $Q_{i}$ denotes the queue of $v_{i}$; $\left|Q_{i}\right|$ is the length of $Q_{i}$; $Q_{i}[r]$ is the r-th packet in $Q_{i}$; and $Q_{i}[r].\;SV$, $Q_{i}[r].\;DV$, $Q_{i}[r].\;SVV$, and $Q_{i}[r].\;FP$ denote the SVF, DVF, SVVF, and FPF of $Q_{i}[r]$ respectively.

The main steps of Algorithm 1 are presented Lines 3–6; this process is known as re-planning. A packet may not be sent in the slots predetermined by its FPF in the limits of FIFO, PIA, and the delivery capability of the vertex. If the current slot is later than the predetermined slot but the packet has not been sent, its FPF becomes invalid immediately. In this case, the vertex at which the packet is located must update the FPF of this packet by replanning. The new FPF is the path whose source vertex is the current vertex, the destination vertex is its DVF, and the planning slot is the current slot.
**Algorithm 1.** Routing decision-making algorithm for a vertex in an IERMN**Input:** Queue of $v_{i}$ and current slot k **Output:** The routing action taken by $v_{i}$ in slot k
c=0**for** r=1 **to** $\left|Q_{i}\right|$ **do****if** $ο(Q_{i}[r].\;FPF)<k$ **then**Obtain $P_{Q_{i}[r].SVF,\;Q_{i}[r].DVF,\;k}$ using a path planning algorithm$Q_{i}[r].FPF=P_{Q_{i}[r].SVF,\;Q_{i}[r].DVF,\;k}$**end if****if** $c<C_{\rho }$ **and** $v_{x_{ik}}\notin Q_{i}[r].\;SVVF$ **and** $(v_{i},e_{i,x_{ik}}^{\left[k\right]},v_{x_{ik}})\subset Q_{i}[r].\;FPF$ **then**$v_{i}$ sends $Q_{i}[r]$ to $v_{x_{ik}}$c←c+1**end if****end for**



## 4. Two Routing Strategies

### 4.1. Shortest Paths in the IERMN

The path planning algorithm is the core of the routing decision-making algorithm for vertices in IERMNs. A routing strategy is defined as a routing decision-making algorithm using a certain path planning algorithm. In this paper, we focus on the shortest path routing strategy in the IERMN, not only because the shortest path routing strategy is the most popular in real-world networks [33], but also because of the characteristic topology of the IERMN.

Since each vertex in an IERMN has, at most, one neighboring vertex in any slot, the IERMN is unconnected at any instant of time. In this case, there are no contemporaneous end-to-end paths between sources and destinations in the IERMN, just like the delay tolerant network [34]. Therefore, many improved routing strategies are unsuitable for the IERMN.

As a path in an IERMN has both a spatial length and a temporal length, there are two kinds of shortest path: one is the Least Delay Path (LDP), which has the shortest temporal length, and the other is the Least Hop Path (LHP), which has the shortest spatial length. There may be more than one LDP or LHP; thus, we are most interested in the Least Delay Path with Minimum Hop (LDPMH) and the Least Hop Path with Minimum Delay (LHPMD). Hence, in this paper, we studied two routing strategies for the IERMN: the LDPMH routing strategy and the LHPMD routing strategy.

### 4.2. Planning Algorithm for the LDPMH

**Figure 2:**
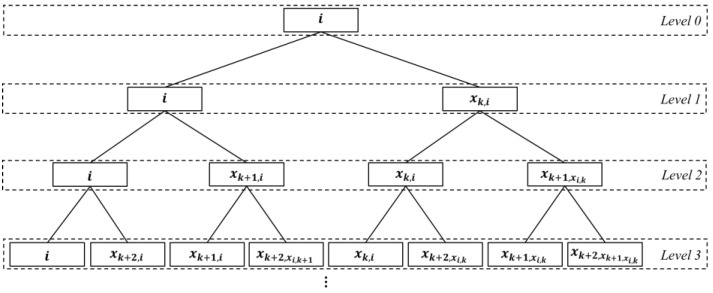
Binary Search Tree for planning the LDPMH. The dotted rectangles indicate the levels in the tree. The solid rectangles indicate the nodes in the tree.

A binary search tree for the planning LDPMH (which is abbreviated to LDPMH-BST) is shown in Figure 2. The node of an LDPMH-BST represents the vertex of an IERMN. In an LDPMH-BST, a parent’s left child is itself, and its right child is the vertex to which it is connected during the corresponding slot. The edge between a parent and its left child indicates that this vertex has decided to refuse to send packets. The edge between a parent and its right child indicates that this vertex has decided to send packets.

The λ-th level of an LDPMH-BST is denoted by LDPMH-BST(λ); the number of the nodes in LDPMH-BST(λ) is denoted by $\left|LDPMH-BST(\lambda )\right|$; the ξ-th node from the left in LDPMH-BST(λ) is denoted by LDPMH-BST(λ,ξ); and the root of the LDPMH-BST is denoted by LDPMH-BST(0,1). The temporal length of the path from the vertex that is represented by LDPMH-BST(0,1) to each vertex that is represented by the node in LDPMH-BST(λ) is λ slots. The planning algorithm for the LDPMH based on the building of an LDPMH-BST is shown in Algorithm 2.
**Algorithm 2.** Planning algorithm for the LDPMH**Input:** X, source vertex $v_{i}$, destination vertex $v_{j}$, planning slot k **Output:** the LDPMH from $v_{i}$ to $v_{j}$
Set i as the root of the LDPMH-BST and create $V^{\prime }=\{v_{i}\}$, the set of vertices that has been added to the LDPMH-BST**for** λ=0 **to** Γ−k **do**Construct a new level LDPMH-BST(λ+1)**for** ξ=1 **to** $\left|LDPMH-BST(\lambda )\right|$ **do****if** $x_{k+\lambda ,\;LDP-BST(\lambda ,\xi )}\neq LDPMH-BST(\lambda ,\xi )$ **do**Set $x_{k+\lambda ,\;LDP-BST(\lambda ,\xi )}$ as the right child of LDPMH-BST(λ,ξ) and LDPMH-BST(λ,ξ) as its left child$V^{\prime }\leftarrow V^{\prime }\cup \left\{v_{x_{k+\lambda ,\;LDPMH-BST(\lambda ,\xi )}}\right\}$**else**Set LDPMH-BST(λ,ξ) as the left child of LDPMH-BST(λ,ξ)**end if****end for****if** $v_{j}\in V^{\prime }$ **then****break****end if****end for****for** each leaf whose value is j **do**Backtrack from the leaf to the root to collect the nodes that have been traveled throughArrange the collected nodes in reverse order to get a sequence that can be transformed into an LDP**end for**Select the sequence with the least different nodes from among the sequences obtained by Steps 16–18 which will be transformed into the LDPMH.



Once the number of the destination vertex, j, has been found in X by Steps 5–7, the construction of the LDPMH-BST stops immediately, according to Steps 12–14. Therefore, the slot that is represented by the level at which the leaves of the LDPMH-BST are is the first to reach $v_{j}$. Then, at least one LDP is obtained by Steps 17–18. After that, the LDPMH can be selected from all of the LDPs.

### 4.3. Planning Algorithm for the LHPMD

**Figure 3:**
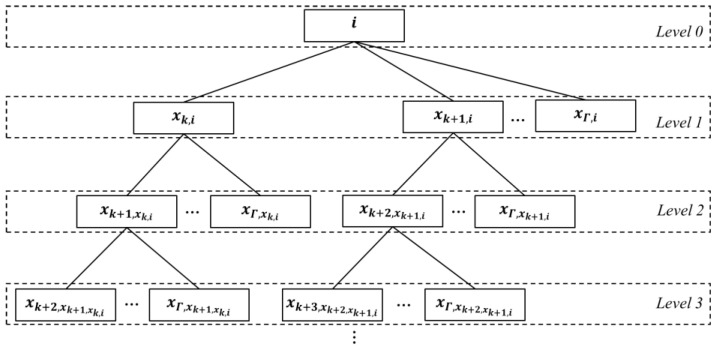
Ordered tree for planning the LHPMD. The dotted rectangles indicate levels in the tree. The solid rectangles indicate nodes in the tree.

An ordered tree for planning LHPMD (which is abbreviated to LHPMD-OT) is shown in Figure 3. The node of an LHPMD-OT also represents the vertex of an IERMN. The λ-th level of an LHPMD-OT is denoted by LHPMD-OT(λ); the number of nodes in LHPMD-OT(λ) is denoted by $\left|LHPMD-OT(\lambda )\right|$; the ξ-th node from the left in LHPMD-OT(λ) is denoted by LHPMD-OT(λ,ξ); and the root of the LHPMD-OT is denoted by LHPMD-OT(0,1). The spatial length of the path from the vertex that is represented by LHPMD-OT(0,1) to each vertex that is represented by the node in LHPMD-OT(λ) is λ hops.

Each node of an LHPMD-OT is designated by a 4-tuple

(13) $$\langle Me,Parent,Level,Number\rangle$$

where Me is the number of the vertex that is represented by the node, Parent is the number of the vertex that is represented by the parent of the node, Level is the level in which the node exists, and Number shows the position of the node in its parent’s children. In this paper, the 4-tuple formula of LHPMD-OT(λ,ξ) is set as $\langle \mu ,\rho ,\lambda ,\iota \rangle$.

The planning algorithm for the LHPMD based on building an LHPMD-OT is shown in Algorithm 3.
**Algorithm 3.** Planning algorithm for the LHPMD**Input:** X, source vertex $v_{i}$, destination vertex $v_{j}$, planning slot k **Output:** the LHPMD from $v_{i}$ to $v_{j}$
Set i as the root of the LHPMD-OT and create $Z=\{v_{i}\}$, the set of nodes of the LHPMD-OT whose value is j.**for** λ=0 **to** Γ **do**Construct a new level LHPMD-OT(λ+1)**for** ξ=1 **to** $\left|LHPMD-OT(\lambda )\right|$ **do****for** τ=k+λ+ι−1 **to** Γ **do**The 4-tuple formula of LHPMD-OT(λ+1,τ) is set as $\langle x_{\tau ,\mu },\mu ,\lambda +1,\tau -k-\lambda -\iota +2\rangle$, because it is the τ-th child of LHPMD-OT(λ,ξ) whose 4-tuple formula is $\langle \mu ,\rho ,\lambda ,\iota \rangle$.**if** $x_{\tau \mu }=j$ **do**Z←Z∪{LHPMD-OT(λ+1,τ)}**end if****end for****end for****if** Z≠∅ **then**Select the nodes with the lowest values of Level from among ZSelect the node with the lowest value of Number from among the nodes selected by Step 13Backtrack from the selected node by Step 14 to the root i to collect the nodes that have been traveled throughArrange the collected nodes in reverse order to get a sequence that will be transformed into the LHPMD**break****end if****end for**



The level of an LHPMD-OT represents the spatial length of the path from the vertex, which is represented by the root, to each vertex, which is represented by the node in it. Hence, a lower level will lead to a path with a smaller spatial length. The children of a node in an LHPMD-OT represent the vertices that are connected by the vertex represented by the node in the future. Thus, a node with a smaller value of Number means that the vertex represented by it will be connected to the vertex represented by its parent earlier. Therefore, by backtracking from the node with the lowest values of Level and Number in Z to the root, the LHPMD can be obtained, according to Steps 13–16.

## 5. Simulation Experiments and Analyses

### 5.1. Simulation Design

Since the vertices of an IERMN are able to control packets and make routing decisions autonomously, the vertices were modeled as agents by the method of Agent Based Modeling and Simulation (ABMS). Then, we conducted traffic dynamics simulations on the System-of-Systems Effectiveness Analysis Simulation (SEAS) platform to compare the two proposed routing strategies. The SEAS is an ABMS platform [35].

We designed eight scenarios to simulate, as tabulated in Table 1. There were differences in the routing strategy, the number of vertices Θ, and the delivery capability C among the eight scenarios. We set the values of C by referring to [33,36].

We performed simulations for each scenario with different packet generation rate R values in order to investigate the value of $R_{C}$. The range of R was set as {1,2,⋯,25}. That is, 25 simulations were conducted for each scenario. Each simulation lasted for 2400 time steps, i.e., 1200 slots. One slot was set as 3 s. The planning time window Γ was set as 20. We created 60,000 random pairs of source and destination, which were chosen sequentially as the SVF and the DVF of each newly generated packet in each simulation.

**Figure 4:**
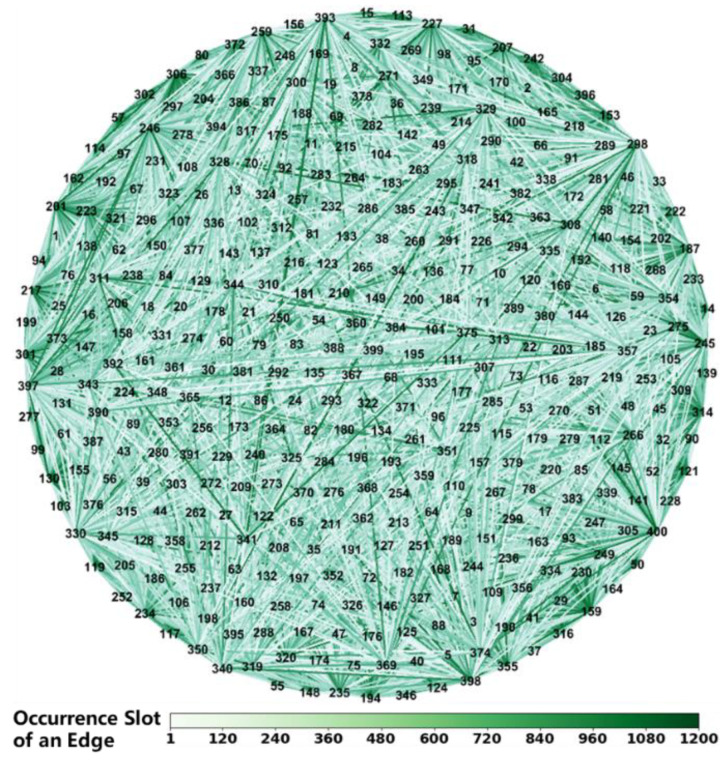
The IERPMN with 400 vertices. The unit of the heat map below is the slot. The numbers in the network diagram above indicate the vertices of the IERPMN. A line between two numbers in the network diagram above indicates the edge connecting these two vertices.

**Figure 5:**
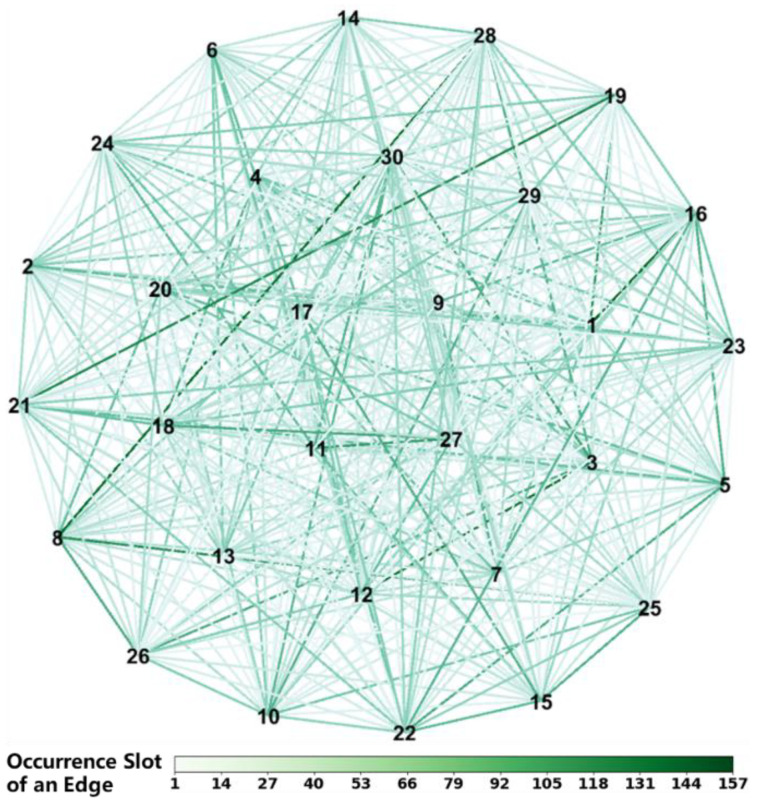
The IERPMN with 30 vertices. The unit of the heat map below is a slot. The numbers in the network diagram above indicate the vertices of the IERPMN. A line between two numbers in the network diagram above indicates the edge connecting these two vertices.

We generated an IERPMN with 400 vertices and an IERPMN with 30 vertices to conduct traffic dynamics simulations, which are shown in Figure 4 and Figure 5. In these two figures, we use numbers rather than symbols to signify vertices. The color of an edge indicates the slot in which it occurs. If the edge occurs in more than one slot, only the first one is considered. These two IERPMNs are based on two real-world networks. The IERPMN with 30 vertices was used to simulate BDS [6]. The IERPMN with 30 vertices was used to simulate one shell of the SpaceX Starlink constellation [37]. In each slot of the IERPMN with 30 vertices, 15 edges were constructed using the method presented in Section 1. The generation of the IERPMN for a slot was independent of those for the other slots.

### 5.2. Simulation Analyses

#### 5.2.1. Simulation Analyses of the Critical Packet Generation Rate

**Figure 6:**
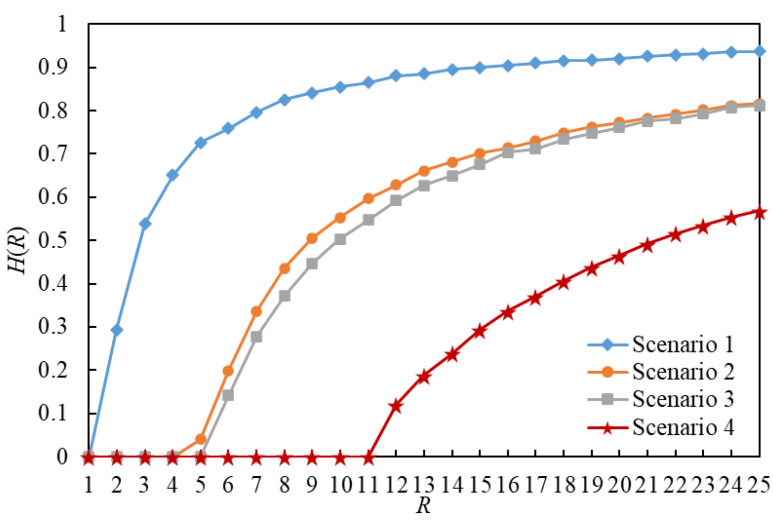
The order parameter *H* versus *R* for Scenarios 1–4. There are $R_{C}(1)\;=\;1$, $R_{C}(2)\;=\;4$, $R_{C}(3)\;=\;5$, and $R_{C}(6)\;=\;11$.

**Figure 7:**
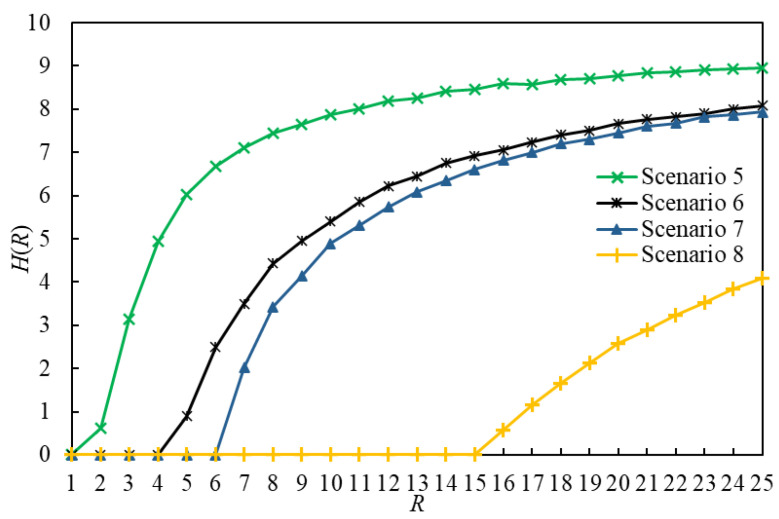
The order parameter *H* versus *R* for Scenarios 5–8. There are $R_{C}(5)\;=\;1$, $R_{C}(6)\;=\;4$, $R_{C}(7)\;=\;6$, and $R_{C}(8)\;=\;15$.

The order parameter H versus R for eight scenarios is illustrated in Figure 6 and Figure 7. In this paper, $R_{C}(1)$ denotes $R_{C}$ in Scenario 1, and so on. By comparing the curves of Scenario $g_{1}$ and Scenario $g_{1}+1$ ($g_{1}\in \{1,3,5,7\}$) in Figure 6 and Figure 7, we found that the value of $R_{C}$ in a scenario using the LDPMH routing strategy is less than that of $R_{C}$ in a scenario using the LHPMD routing strategy under the same conditions. Thus, it is easier for the LDPMH routing strategy to cause traffic congestion in an IERMN than it is for the LHPMD routing strategy.

The reason for this phenomenon is that the planning costs of the LDPMH and LHPMD are different. An LDPMH is planned at the expense of a larger spatial length so that the LDPMH may have the shortest temporal length. On the contrary, an LHPMD is planned at the expense of a larger temporal length so that the LHPMD may have the shortest spatial length. Therefore, the LDPMH is assumed to contain more vertices than the LHPMD. If all paths of all packets contain quantities of vertices, each vertex has to transmit a large number of packets. The high load of vertices must induce traffic congestion. Hence, $R_{C}$ by the LDPMH routing strategy is less than $R_{C}$ by the LHPMD routing strategy.

#### 5.2.2. Simulation Analyses of the Number of Delivered Packets

**Figure 8:**
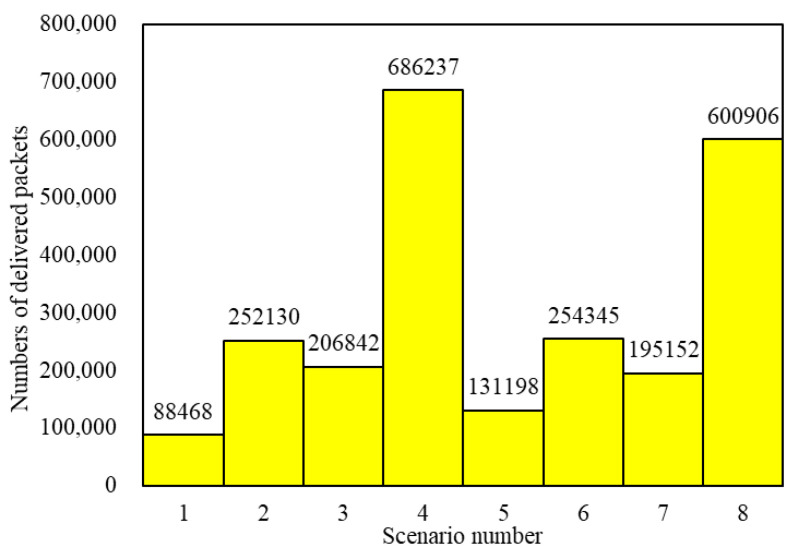
The numbers of delivered packets in the 8 scenarios.

A total of 2,419,434 packets were delivered through 8×25=200 simulations in 8 scenarios. The numbers of delivered packets in the 8 scenarios are illustrated in Figure 8. Obviously, a scenario using the LHPMD routing strategy will have more delivered packets than a scenario using the LDPMH routing strategy under the same conditions.

**Figure 9:**
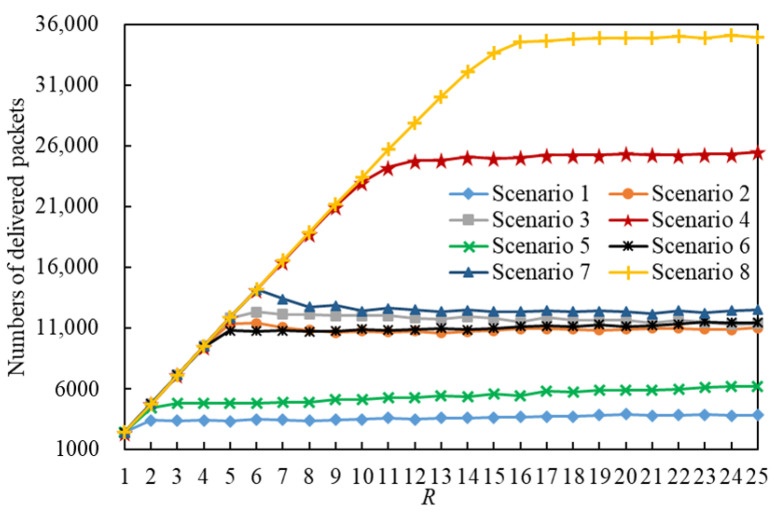
The number of delivered packets versus *R*.

We analyzed the number of delivered packets in each scenario. Figure 9 shows the relationship between the number of delivered packets and R. If we compare the curves of Scenario $g_{1}$ and Scenario $g_{1}+1$ ($g_{1}\in \{1,3,5,7\}$) presented in Figure 9, we find that:

1. When $R\leq R_{C}(g_{1})$, the IERMNs in Scenario $g_{1}$ and Scenario $g_{1}+1$ are in the free-flow state and there are tiny differences in the number of delivered packets between them.
2. When $R>R_{C}(g_{1})$, the IERMNs in Scenario $g_{1}$ and Scenario $g_{1}+1$ are in the congestion state and there are large gaps in the number of delivered packets between them.

**Figure 10:**
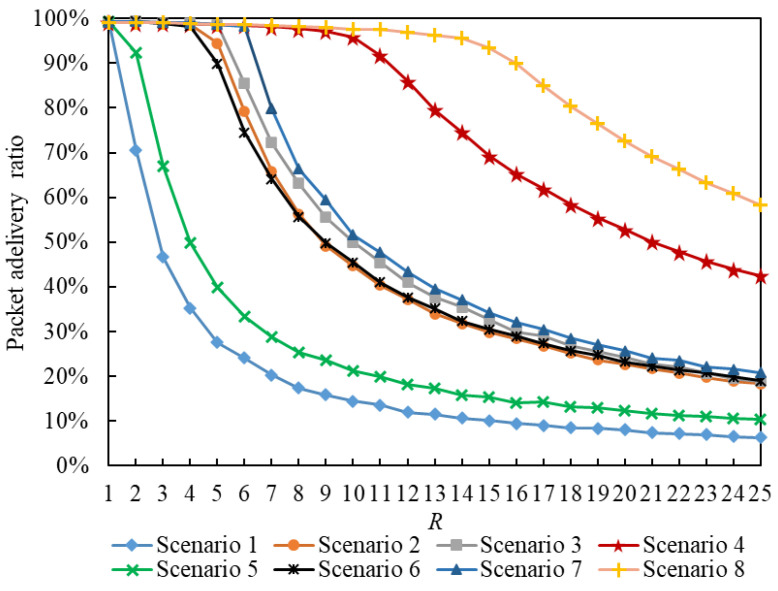
The packet delivery ratio versus *R*.

Figure 10 shows the relationship between the packet delivery ratio and R. The packet delivery ratio [38] is the proportion of delivered packets from all packets. If we compare the curves of Scenario $g_{1}$ and Scenario $g_{1}+1$ ($g_{1}\in \{1,3,5,7\}$) in Figure 10, we find that when $R>R_{C}$, i.e., the IERMNs become more congested with an increase in R, the packet delivery ratios in a scenario using the LDPMH routing strategy decrease more rapidly than those in a scenario using the LHPMD routing strategy under the same conditions.

Therefore, when in the free-flow state, an IERMN using the LDPMH routing strategy and one using the LHPMD routing strategy have almost the same number of delivered packets; when in the congestion state, an IERMN using the LHPMD routing strategy has more delivered packets than the IERMN using the LDPMH routing strategy.

#### 5.2.3. Simulation Analyses of the Posterior Path Length

**Figure 11:**
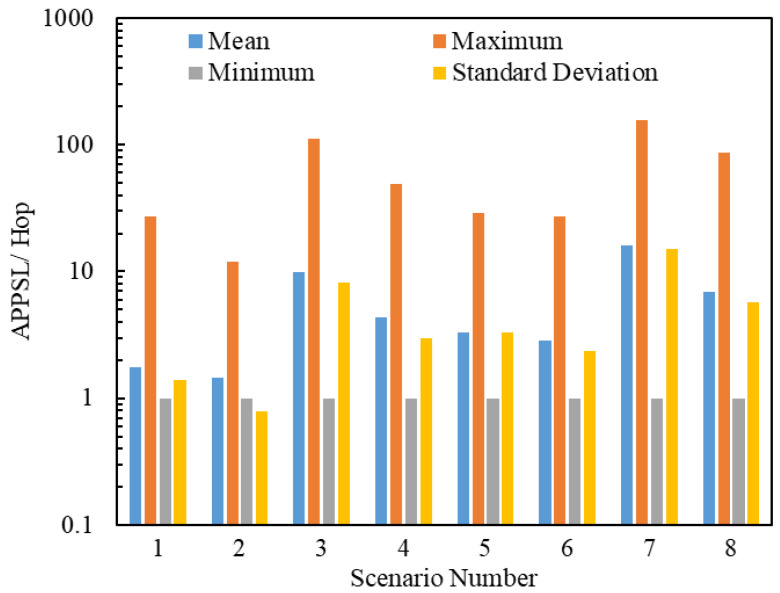
Statistics on the APPSLs for Scenarios 1–8. The ordinate scale uses a logarithmic scale.

**Figure 12:**
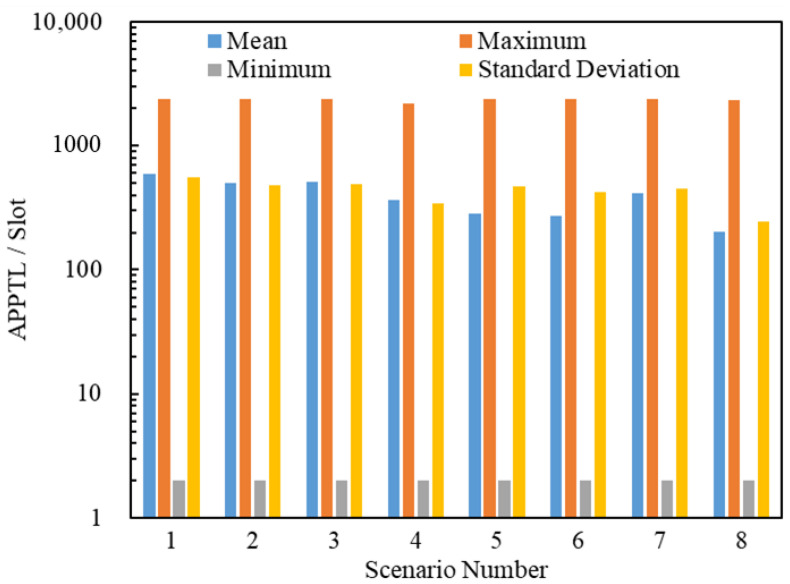
Statistics on the APPTL s for Scenarios 1–8. The ordinate scale uses a logarithmic scale.

In this section, we present the analysis of the average posterior path lengths of 2,419,434 delivered packets. Figure 11 and Figure 12 illustrate the statistics on the Average Posterior Path Spatial Lengths (APPSLs) and the Average Posterior Path Temporal Lengths (APPTLs). The eight scenarios have the same minimum APPSL; they also have the same minimum APPTL. Scenario $g_{1}$ has larger means, maximums, and standard deviations for the APPSL and APPTL than Scenario $g_{1}+1$ ($g_{1}\in \{1,3,5,7\}$). In general, a scenario using the LHPMD routing strategy has smaller posterior path lengths than a scenario using the LDPMH routing strategy under the same conditions. We analyzed the average posterior path lengths of the delivered packets in each scenario.

**Figure 13:**
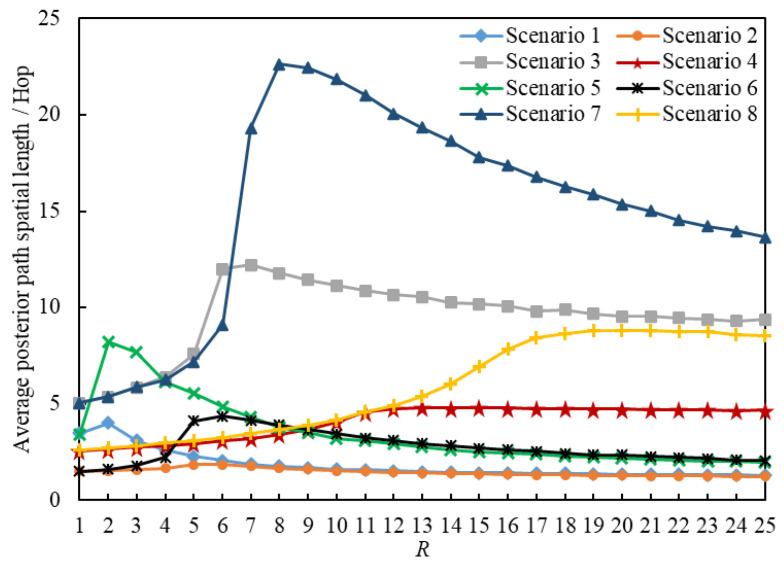
Average posterior path of the spatial length versus *R*.

**Figure 14:**
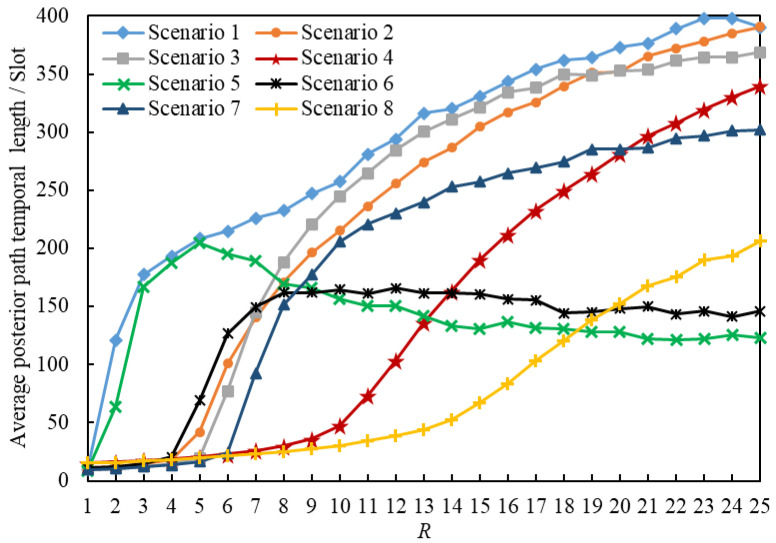
Average posterior path of the temporal length versus *R*.

Figure 13 illustrates the relationships between the APPSLs of the delivered packets and R. Figure 14 illustrates the relationships between the APPTLs of the delivered packets and R. If we compare the curves of Scenario $g_{1}$ and Scenario $g_{1}+1$ ($g_{1}\in \{1,3,5,7\}$) presented in Figure 13 and Figure 14, we find that: when $R\leq R_{C}(g_{1})$, the APPSLs in a scenario using the LDPMH routing strategy overwhelm those in a scenario using the LHPMD routing strategy under the same conditions, while the difference in their APPTLs is quite small. Hence, when an IERMN is in the free-flow state, it has a smaller average posterior path length for the delivered packets when the LHPMD routing strategy is used compared with the LDPMH routing strategy.

Nevertheless, when the IERMN is in the congestion state, there are different performances in different scenarios, which can be described as follows.

1. When $R>R_{C}(2)$, the gap between Scenario 1 and Scenario 2 in the APPSL is narrowed with an increase in R, and the APPSLs of Scenario 1 are always larger than those of Scenario 2, so the APPTLs are too.
2. When $R>R_{C}(6)$, the APPSLs and the APPTLs of Scenario 5 are larger than those of Scenario 6 in the beginning; afterwards, Scenario 6 overtakes Scenario 5 as R increases.
3. When $R\leq R_{C}(g_{2})$ ($g_{2}\in \{3,7\}$), the gap between Scenario $g_{2}+1$ and Scenario $g_{2}$ in terms of the APPSLs becomes a little smaller, and the APPSLs of Scenario $g_{2}+1$ outnumber those of Scenario $g_{2}$, so the APPTLs do too.

When the IERMN is congested, in terms of the average posterior path length, a path with more vertices or fewer vertices with a poor delivery capability will have a shorter average posterior path length for the packets delivered by the LHPMD routing strategy than those delivered by the LDPMH routing strategy; if a path has fewer vertices with a strong delivery capability, it will have a shorter average posterior path length for the delivered packets with the LDPMH routing strategy than with the LHPMD routing strategy.

The reason why the LHPMD routing strategy leads to a smaller APPTL in some cases is also due to the different planning costs of an LDPMH and an LHPMD. As mentioned in Section 5.2.1, the LDPMH should contain more vertices than the LHPMD. Within the limits of the FIFO, PIA, and C, the more vertices a packet visits, the more frequently the packet may be held. Therefore, it may take more time to transmit the packet with LDPMHs than with LHPMDs.

### 5.3. Comparison with the Benchmark Method

In this section, the performance of the proposed routing strategies is compared with a benchmark method called the flooding routing strategy, which means that each vertex always sends C packets to its successor vertex until the packet has reached its destination. We designed 4 scenarios using the flooding routing strategy, as tabulated in Table 2. The simulations for Scenarios 9–12 were designed in the same way as Scenarios 1–8, as presented in Section 5.1.

**Figure 15:**
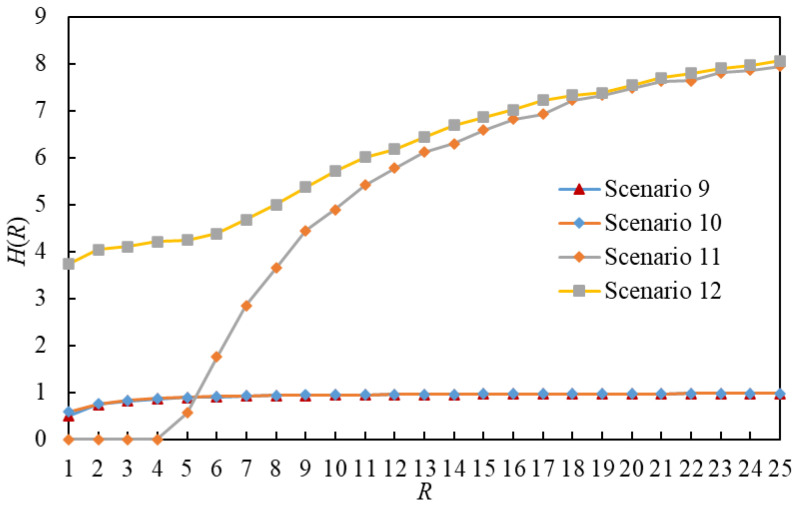
The order parameter *H* versus *R* for Scenarios 9–12. There are $R_{C}(9)\;=\;0$, $R_{C}(10)\;=\;1$, $R_{C}(11)\;=\;0$ and $R_{C}(6)\;=\;0$.

A comparison of the order parameter H versus R for eight scenarios is illustrated in Figure 15. By comparing Figure 15 with Figure 6 and Figure 7, we find that $R_{C}$ in a scenario using the flooding routing strategy is more than in a scenario using the LHPMD or LDPMH routing strategy under the same conditions. Thus, in terms of the critical packet generation rate, the flooding routing strategy has the worst performance among these three routing strategies.

**Figure 16:**
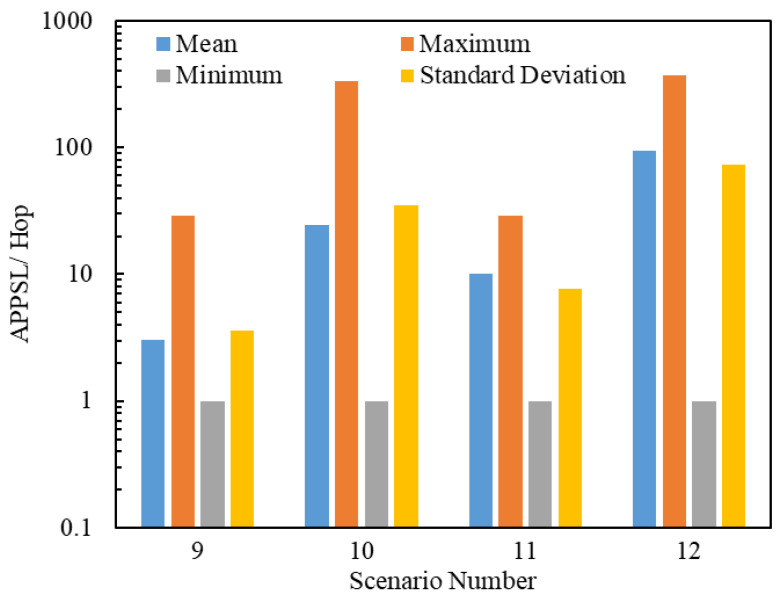
Statistics on the APPSL s of Scenarios 9–12. The ordinate scale uses a logarithmic scale.

**Figure 17:**
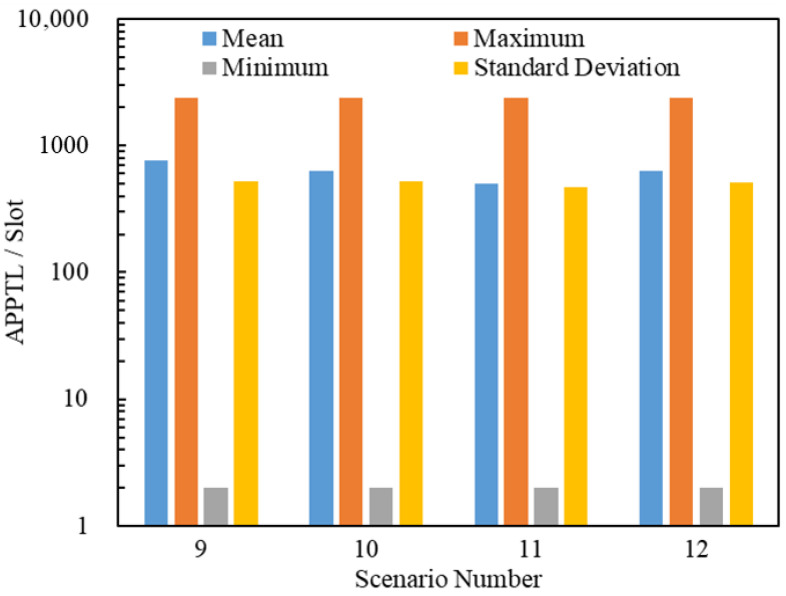
Statistics on the APPTL s of Scenarios 9–12. The ordinate scale uses a logarithmic scale.

A total of 560,139 packets were delivered through 4×25 = 100 simulations by 8 scenarios. Figure 16 and Figure 17 illustrate the statistics on the APPSLs and APPTLs of the 560,139 delivered packets. By comparing Figure 16, Figure 17, Figure 11 and Figure 12, we find that a scenario using the flooding routing strategy has larger means for the APPSL and APPTL than a scenario using the LHPMD or LDPMH routing strategies under the same conditions. The dispersion of the APPSLs and APPTLs for a scenario using the flooding routing strategy is also large in terms of the minimum, maximum and standard deviation values. Thus, in terms of the average posterior path lengths, the flooding routing strategy has the worst performance among these three routing strategies.

## 6. Discussion and Conclusions

In this paper, we put forward a new network model called an IERMN and two routing strategies for the IERMN: the LDPMH routing strategy and the LHPMD routing strategy. We drew some conclusions about the two proposed routing strategies by the traffic dynamics simulations, which are described as follows:

1. In terms of the maximum traffic capability of the IERMN, the LHPMD routing strategy performs better than the LDPMH routing strategy.
2. In terms of the capability of delivering packets to their destinations, when the IERMN is in the free-flow state, the performance of the LDPMH routing strategy nearly equals that of the LHPMD routing strategy; when the IERMN is congested, the LHPMD routing strategy overwhelms the LDPMH routing strategy.
3. In terms of the average posterior path length of the delivered packets, when the IERMN is in the free-flow state, the LHPMD routing strategy is better than the LDPMH routing strategy; when the IERMN is in the congestion state, the LHPMD routing strategy is better in some cases, and the LDPMH routing strategy is better in other cases.

Although the LDPMH routing strategy leads to a shorter average posterior path length for the delivered packets in some cases, it results in fewer delivered packets, which is worthwhile. On the whole, the LHPMD routing strategy outperforms the LDPMH routing strategy. Regardless of whether an IERMN is in the free-flow state or in the congestion state, the LHPMD routing strategy is always recommended.

Future research surrounding the IERMN should include its evolution direction, its spread dynamics, and its synchronization dynamics. These are our future aims and we hope that more and more researchers will study IERMNs.

## Figures and Tables

**Table 1:** Setup in scenarios.

Scenario Number	Routing Strategy	*Θ*	*C*
1	LDPMH	30	1
2	LHPMD	30	1
3	LDPMH	400	1
4	LHPMD	400	1
5	LDPMH	30	10
6	LHPMD	30	10
7	LDPMH	400	10
8	LHPMD	400	10

**Table 2:** Setup in scenarios using the flooding routing strategy.

Scenario Number	Routing Strategy	*Θ*	*C*
9	Flooding	30	1
10	Flooding	400	1
11	Flooding	30	10
12	Flooding	400	10

## Data Availability

Not applicable.

## Abbreviation

**A** **bbreviation**	**Meaning**
ABMS	Agent Based Modeling and Simulation
APPSL	Average Posterior Path Spatial Length
APPTL	Average Posterior Path Temporal Length
BDS	BeiDou Navigation Satellite System
LDPMH-BST	Binary Search Tree for planning LDPMH
DVF	Destination Vertex Field
FIFO	First-In-First-Out
FPF	Future Path Field
ISL	Inter-Satellite Links
IEN	Isochronal-Evolution Networks
IERMN	Isochronal-Evolution Random Matching Network
IERPMN	Isochronal-Evolution Random Perfect-Matching Network
LDP	Least Delay Path
LDPMH	Least Delay Path with Minimum Hop
LHP	Least Hop Path
LHPMD	Least Hop Path with Minimum Delay
MCN	mobile call network
LHPMD-OT	Ordered Tree for planning LHPMD
PIA	Path Iteration Avoidance
RMN	Random Matching Network
RPMN	Random Perfect-Matching Network
SVVF	Set of Visited Vertices Field
SVF	Source Vertex Field
SEAS	System-of-systems Effectiveness Analysis Simulation
**Notation**	**Meaning**
Θ	number of all vertices in a network
θ	number of all edges in a network
X	matrix that shows which edges exist at some slot
i	vertex number
j	vertex number
$v_{i}$	vertex whose number is i
k	slot number
$\begin{array}{c} x_{ki} \end{array}$	vertex that is connected by *v_i_* in slot *k*
$e_{ij}^{\left[k\right]}$	edge between *v_i_* and *v_j_* in slot *k*
H	order parameter to characterize the congestion phase transition
R	packet generation rate
C	delivering capability of a vertex
$P_{ijk}$	path which is from $v_{i}$ to $v_{j}$ and planned at slot k
$\upsilon (P_{ijk})$	departure slot of $P_{ijk}$
$ο(P_{ijk})$	arrival slot of $P_{ijk}$
Γ	planning time window
$Q_{i}$	queue of $v_{i}$
$\left|Q_{i}\right|$	ength of $Q_{i}$
$Q_{i}[r]$	r-th packet in $Q_{i}$
λ	level number of an LDPMH-BST or an LHPMD-OT
ξ	Node number in a level of an LDPMH-BST or an LHPMD-OT
$V^{\prime }$	set of vertices that have been added in a LDPMH-BST
Z	set of nodes of the LHPMD-OT whose value are j
$g_{1}$	scenario number
